# Effect of isometric handgrip exercise on cognitive function: Current evidence, methodology, and safety considerations

**DOI:** 10.3389/fphys.2022.1012836

**Published:** 2022-10-04

**Authors:** Yuxin Zhu, Shan He, Fabian Herold, Fenghua Sun, Chunxiao Li, Sisi Tao, Tian-Yu Gao

**Affiliations:** ^1^ School of Physical Education, Guangzhou Sport University, Guangzhou, China; ^2^ Department of Rehabilitation Sciences, The Hong Kong Polytechnic University, Hong Kong SAR, China; ^3^ Research Group Degenerative and Chronic Disease, Movement, Faculty of Health Sciences Brandenburg, University of Potsdam, Potsdam, Germany; ^4^ Department of Health and Physical Education, The Education University of Hong Kong, Hong Kong SAR, China; ^5^ School of Physical Education and Sports Science, South China Normal University, Guangzhou, China; ^6^ Faculty of Education, The University of Hong Kong, Hong Kong SAR, China; ^7^ School of Physical Education, Jinan University, Guangzhou, China

**Keywords:** systematic review, cognition, executive function, clench, static exercise

## Abstract

Cognitive function is essential for most behaviors of daily living and is a critical component in assessing the quality of life. Mounting prospective evidence supports the use of isometric handgrip exercise (IHE) as a small muscle mass practice to promote health-related outcomes in clinical and healthy populations. The aim of the present review was to systematically investigate whether IHE is effective in improving the cognitive function of adults (aged ≥18 years). Studies were identified by searching five databases (CINAHL, MEDLINE, SPORTDiscus, PsychINFO, and Web of Science). Eight out of 767 studies met the inclusion criteria, including three types of studies: 1) acute effect for IHE with various intensity protocols (*n* = 4); 2) acute effect for IHE with one set exhaustion protocol (*n* = 2); and 3) chronic effect of IHE on cognitive function (*n* = 2). To assess the methodological quality of studies, the PEDro scale was used (mean score = 6.75). The evidence on whether IHE exerts acute positive effects on cognitive performance is currently rather inconclusive. However, a trend was discernible that implementing IHE can generate a beneficial chronic effect on cognitive function, although the results should be interpreted with caution. The clinical relevance of IHE as a time-efficient type of physical exercise to improve cognitive function warrants further investigation. Methodology and safety considerations were discussed.

**Systematic Review Registration**: (https://osf.io/gbzp9).

## 1 Introduction

Cognitive function is critical for most daily living behaviors and essential to health-related quality of life (Pusswald et al., 2015). After the age of 30 years, the whole-brain volume decreases by 0.45% each year in healthy populations, increasing the risk of progressing to mild cognitive impairment and dementia (Fotenos et al., 2005). Understanding the factors that maintain or enhance cognitive function is a common goal in health sciences and related disciplines. Physical exercise, particularly resistance exercise (RE), has recently been recommended as the most effective type of exercise for improving cognition (Huang et al., 2021). RE contributes to the maintenance and increase of muscle strength and mass, triggering positive neurobiological processes and being critical for preserving brain and cognitive functions (Herold et al., 2019b). Of the two types of RE, dynamic RE (i.e., movements that require the muscles to resist weight over a range of motion) is found to have a small to moderate positive effect on cognitive function in healthy and cognitively impaired adults (Wilke et al., 2019; Huang et al., 2021). Isometric RE (i.e., the application of muscle force without the movement of a joint), however, has received relatively less attention because it had been found to be related to increased systolic blood pressure (SBP) and diastolic blood pressure (DBP), which in turn increased the potential risk of adverse events such as heart disease and stroke in participants during exercise (Huggett et al., 2004).

Over the last decades, however, research into isometric RE has evolved and the potential safety issues have been studied. The current evidence suggests that isometric exercises are relatively safe (Olherdos et al., 2013; Nielson et al., 2014; Hansford et al., 2021; Baffour-Awuah et al., 2022), given that only one adverse event occurs for every 38,444 isometric RE performed (Hansford et al., 2021). Notably, isometric RE has been classified as one of the best non-pharmacological interventions for preventing and treating hypertension in the 2017 American College of Cardiology and American Heart Association guidelines (Whelton et al., 2018). There is increasing evidence that chronic isometric RE leads to significant reductions in resting blood pressure (BP) in hypertensive and normotensive men and women (Carlson et al., 2014; Hess and Smart, 2017), and the magnitude is greater than the effects of dynamic RE, aerobic exercise (Carlson et al., 2014), high-intensity interval training (Edwards et al., 2022) and is comparable to the effect of beta-blockade monotherapy (Wong and Wright, 2014). A close relationship between BP and cognitive function has implications for global health care (Novak and Hajjar, 2010; Forte et al., 2020). Abnormal BP (i.e., hypertension and hypotension) leads to decreases in perfusion, oxygenation, and vascular reserve capacity, which have been associated with declines in cognitive function as well as dementia (Novak and Hajjar, 2010). In addition, it may accelerate the age-related decline in blood flow and brain tissue volume and have an additive effect on worsening cognitive outcomes in later life (Novak and Hajjar, 2010). Based on the evidence and potential mechanism mentioned above, isometric RE appears to be a safe and promising intervention strategy to foster healthy cognitive aging, although the current evidence in this direction has not been systematically analyzed.

Isometric handgrip exercise (IHE) is a form of isometric RE that involves only a small amount of muscle and does not require a full-body workout. It is easier to implement in populations for whom larger muscle mass exercises are more challenging or not feasible, such as older adults and patients exercising in certain locations (e.g., hospitals) or circumstances (e.g., bedridden patients). Generally, the appropriate level of exercise-induced arousal positively affects cognitive performance (Huang et al., 2021). However, evidence-based studies have shown that the effects of IHE on cognitive function are currently inconclusive. For example, Washio et al. (2021) found that after two mins of IHE at 25% of maximum strength, post-exercise cognitive processing speed improved compared with the control group. However, Yamada et al. (2021) found no significant difference in cognitive performance (measured by the Stroop task [ST]) in healthy adults after four sets of two mins IHE at 30% maximum strength, compared with a control group. Brown and Bray (2015) observed a dose-response relationship between IHE and cognitive performance, with those who performed the IHE to exhaustion showing a decrease in cognitive performance (measured by ST). The inconsistent results may be due to methodological differences concerning exercise variables (e.g., exercise intensity, duration) and the specific domain of cognition measured (Lambourne and Tomporowski, 2010). Therefore, a systematic review is warranted to synthesize the mixed findings and provide suggestions for future studies.

To our best knowledge, one review study has summarized the association between IHE and health-related outcomes, which included cognition as an outcome (Yamada et al., 2022). However, the conclusion on the effects of IHE on cognitive function was rather preliminary; the majority of included studies focused on the simultaneous episodic memory when performing IHE contractions, and only one pilot study investigated the training effect of IHE on cognitive function in that review. Whether there is an acute/chronic effect after repeated IHE sessions on distinct cognitive function is largely unknown. Other related review studies have indicated that handgrip strength is an indicator of overall muscle strength that can be used in clinical and epidemiological settings for helping to determine the onset and progress of cognitive impairment (Fritz et al., 2017; McGrath et al., 2018, 2020; Shaughnessy et al., 2020; Zammit et al., 2021; Kunutsor et al., 2022). As an increasing number of empirical studies have been conducted in recent years, a summary of the current findings with an analysis of the methodological and safety issues will be an aid for future studies. The present review aims to address the question of whether the cognitive function can be improved acutely and chronically after IHE. The results are expected to support the development of evidence-based public health guidelines aimed at preventing or attenuating the progression of Alzheimer’s disease in specific populations. (e.g., bedridden older adults or adults with serve mobility impairments).

## 2 Materials and methods

### 2.1 Protocol and registration

The current protocol followed the instruction of the PRISMA systematic review checklist (Page et al., 2021). The protocol for this systematic review was registered on OSF Registries (https://osf.io/gbzp9).

### 2.2 Search strategy and eligibility criteria

Five databases were systematically searched in March and updated in August 2022: Ebcohost (CINAHL, MEDLINE, SPORTDiscus, PsychINFO) and Web of science. The snowballing strategy was used to search the reference list of involved studies. Two groups of keywords were used to identify potential studies ([isometric resistan* OR isometric strength OR static resistan* OR static strength] AND [cognitive function OR cognition OR executive function]). Studies were loaded into a reference managing software (Mendeley, version 1.19.8, Mendeley Ltd., London, United Kingdom) that automatically removed existing duplicates. Two independent reviewers (Z.Y and H.S) screened the title and abstract from the searched studies. Any discrepancies were requested by the third reviewer to reach an agreement (S.F).

The inclusion criteria of the studies were 1) participants were individuals with age above 18 years old; 2) experimental study investigated the effects of isometric RE (contraction time longer than five seconds) on cognitive function; 3) exercise modalities adopted by studies were carried out in the form of handgrip activity; 4) original research study was written in English. The studies were excluded if they were 1) animal or artificial research; 2) cross-sectional and qualitative studies; 3) were not original/empirical studies, such as book review and conference presentation; and 4) the purpose of the study was not investigating the acute (short-term)/chronic (long-term) cognitive function performance following isometric handgrip exercise (e.g., transients isometric muscle contractions affect visual attention and inhibitory control; isometric force control during single and dual-task conditions).

### 2.3 Data extraction

Data were extracted by the first author (Z.Y) and checked for consistency by another author (H.S). Specific information for individual studies was extracted, such as author names, publication years, country for the study (if the experiment was conducted in a specific country), study design, sample size, the mean age of participants, intervention-related methodological details, blood pressure responses, cognitive function assessments, and key findings.

### 2.4 Assessment of risk of bias

The risk of bias was assessed using the 11 items Physiotherapy Evidence Database (PEDro) scale (PEDro scale, 1999). Each satisfied item (except item one) contributes one point to the total score, with six or higher indicating a critical point for good to the excellent quality of the studies’ methodology (Cashin and McAuley, 2020). Two reviewers (Z.Y and H.S) independently quantified the scores of each study, with disagreements resolved by the third reviewer (S.F).

## 3 Result

### 3.1 Study selection

**FIGURE 1:**
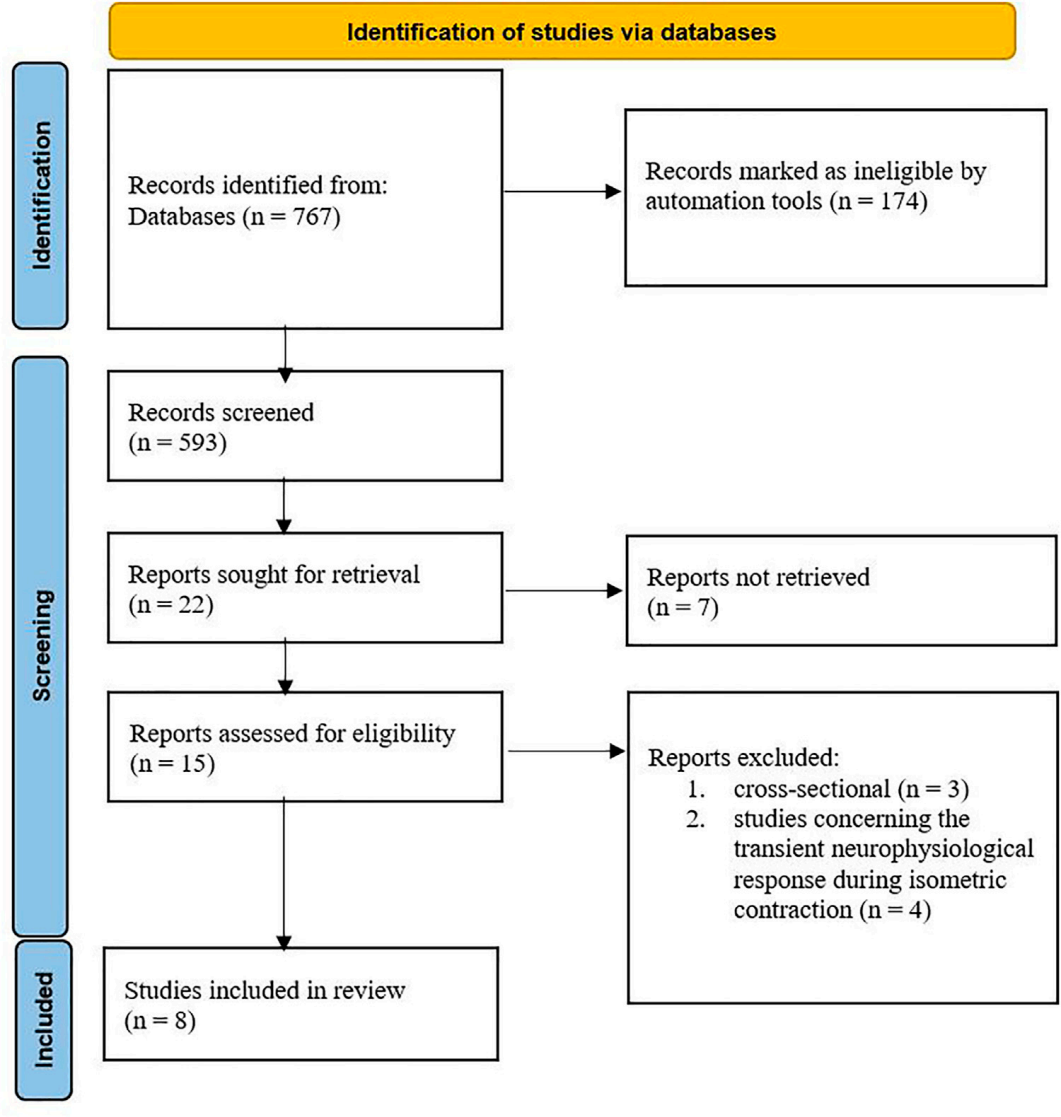
PRISMA diagram.

After removing the duplicates, 22 studies were identified. After screening the titles and abstracts, seven studies were excluded from the review, with 15 studies potentially meeting the inclusion criteria. After screening the full text by two reviewers, seven were excluded for two reasons: 1) cross-sectional studies or 2) studies concerning the transient neurophysiological response during isometric contraction. Finally, eight studies were eligible and included in the current review. The search and screening process was conducted according to the PRISMA flowchart shown in Figure 1.

### 3.2 Risk of bias assessment

The quality of the studies was rated between four and eight (mean = 6.75, see Table 1). The lower score is related to the fact that the study did not specify a blinding strategy and the nature of the exercise-based intervention may not have allowed for true blinding. Furthermore, since Demsper et al. (2018) was a pilot study without a control group, the quality of the study was rated as inadequate. The appraisal of each item of the studies can be found in Supplementary Table S1.

**TABLE 1:** Extracted data from included studies.

First author	Participants characteristics	Study design	Intervention characteristics	Blood pressure responses (mmHg)	Cognition assessment	Key findings	Quality
Washio et al., 2021 Japan	*N* = 17	Crossover	*Exp:* 4 sets; 2-min 25% MVC with 3-min recovery	^#^SBP _rest_ = 124.2 ± 14.1^#^DBP _rest_ = 71.7 ± 6.4	Memory recognition task & Go/No-Go task	Acute effect of IHE improved the processing speed (reaction time) in executive function (Go/No-Go task)	7
	M _age_ = 21.6		*Con:* Waiting list	SBP _exercise_ = 154.0 ± 19.7DBP _exercise_ = 91.7 ± 9.3			
	Male = 82%			SBP _recovery_ = 123.7 ± 13.6DBP _recovery_ = 70.9 ± 7.0			
Yamada et al., 2021 U. S	*N* = 60	Crossover	Exp *1:* 4 sets; 2-min 30% MVC with 1-min recovery; with 50% blood flow restriction	None	Stroop task	Acute effect of a single bout of IHE did not improve the Stroop task performance in two conditions	7
	M _age_ = 21.8		Exp *2:* 4 sets; 2-min 30% MVC with 1-min recovery; without blood flow restriction				
	Male = 35%		*Con:* Waiting list				
Saito et al., 2021 Japan	*N* = 22	Crossover	*Exp:* 16 sets, 30-s 30% MVC with 45-s recovery	*Exp*	Memory recognition task & Go/No-Go task	Acute effect of a single bout of IHE did not improve the cognitive performance	7
	M _age_ = 22		*Con:* Waiting list (read a magazine)	SBP = 116 ± 7/127 ± 11^*^			
	Male = 82%			DBP = 67 ± 5/76 ± 9^*^			
				*Con*			
				SBP = 116 ± 12/118 ± 11			
				DBP = 69 ± 7/71 ± 8			
Mather et al., 2021 U. S	*N* = 87	RCT	*Exp:* 5 sets, 18-s 100% MVC with 60-s recovery	None	Auditory oddball task	Acute effect of maximal isometric ball squeeze exercise led to phasic arousal responses to target-relevant stimuli and improved attentional performance	7
	M _age young_ = 21.2		*Con:* Waiting list (hold the ball but don't squeeze it)				
	M _age old_ = 62.5						
	Male = 0%						
Brown and Bray., 2015 Canada	*N* = 55	RCT	Three intensities until failure	None	Modified Stroop task	Acute effect of performing IHE to exhaustion is associated with impaired cognitive performance. Higher intensity IHE leads to greater performance impairments in a linear dose-response manner	7
	M_age_ = 20.58		*Exp:* N = 14, 30% MVC; N = 13, 50% MVC; N = 14, 70% MVC				
	Male = 40%		*Con:* N = 14				
			5 N IHE				
Guzmán-González et al., 2020 Chile	*N* = 12	Crossover	50% MVC until failure under cognitive task	None	Mathematical task (-7 from a random number between 300 and 700)	The cognitive performance was decreased during the regulated dual-task but not in the self-regulated dual-task or control condition	7
	M age = 20		Exp *1*: Self-regulated dual-task (own pace)				
	Male = 100%		Exp *2*: Regulated dual-task (imposed pace)				
			*Con*: Without cognitive load				
Dempster et al., 2018 Canada	*N* = 8	RCT (Pilot)	8-weeks	*Exp*	Trail making test (Part A and Part B) & Controlled oral word association task	Chronic effect of IHE reduced the time spent on Trial making test part A	4
	M _age_ = 61		*Exp:* 4 sets; 2-min 30% MVC with 60-s recovery, 3 days/week	SBP = 132 ± 4/128 ± 4^*^			
	Male = 63%		*Con:* None	DBP = 85 ± 3/81 ± 2			
			Compliance: 96.6%				
Okamoto and Hasimotto., 2022 Japan	*N* = 22	Quasi-experimental	8-weeks	*Exp*	Trail making test (Part A and Part B)	Chronic effect of IHE reduced the time spent on Trial making test part A and part B	8
	M _age_ = 75		*Exp*	SBP = 139 ± 5/130 ± 4^*,†^			
	Male = 59%		*N* = 11	DBP = 79 ± 3/77 ± 2			
			4 sets; 2-min	*Con*			
			30% MVC with 60-s recovery	SBP = 139 ± 2/140 ± 3			
			5 days/week	DBP = 79 ± 2/78 ± 2			
			*Con*: Waiting list				

Notes: RCT, randomized controlled trial; Exp, Experimental group; Con, Controlled group; MVC, maximal voluntary contraction; SBP, systolic blood pressure; DBP, diastolic blood pressure; IHE, isometric handgrip exercise. ^#^ Total sample, no comparison was made between groups; ^*^ Significant (*p* < 0.05) difference from the baseline; ^†^ Significant (*p* < 0.05) difference from the control.

### 3.3 Participants’ characteristics and study designs

The reviewed studies included four randomized crossover-controlled design studies, two randomized controlled design studies, one randomized pilot study and one quasi-experimental study. The studies were published from 2015 to 2022 and were carried out in four countries, i.e., the U.S, Canada, Chile, and Japan. The reviewed studies involved 283 participants with a mean age of 35.48 years. Participants involved in the studies were healthy adults. The percentage of gender was roughly balanced (male = 57.63%). Six studies investigated the acute effect of a single bout of isometric handgrip exercise on subsequent cognitive performance, including two studies that conducted an IHE until exhaustion. The remaining two studies examined the chronic effects of an eight-week IHE on cognitive function. The extracted data from the studies are presented in Table 1.

### 3.4 Intervention characteristics

The varying intensity of IHE was administered in the included studies and a detailed overview is provided in Table 1. Regarding studies that adopted exhaustion exercise protocol, one study used 30%, 50%, and 70% maximal voluntary contraction (MVC) to perform IHE until exhaustion (Brown and Bray, 2015), and the other study performed the 50% MVC until exhaustion (Guzmán-González et al., 2020). Of the remaining six studies, four sets were conducted in a single session of IHE in four studies (Dempster et al., 2018; Okamoto and Hashimoto, 2022; Washio et al., 2021; Yamada et al., 2021). Two studies used 16 sets (Saito et al., 2021) and five sets (Mather et al., 2020), respectively.

With respect to the holding time, four studies used a holding time of two mins (Dempster et al., 2018; Okamoto and Hashimoto, 2022; Washio et al., 2021; Yamada et al., 2021). Of these studies, one used 25% MVC with three mins recovery (Washio et al., 2021); three were performed on 30% MVC with one min recovery (Dempster et al., 2018; Okamoto and Hashimoto, 2022; Yamada et al., 2021). In one study the holding time lasted 30-s with 30% MVC and 45-s recovery (Saito et al., 2021), whereas one study used a holding time of 18-s at 100% MVC with 60-s recovery (Mather et al., 2020), respectively.

#### 3.4.1 Unilateral or bilateral hands

In two of the studies reviewed, unilateral contractions were used alternately when performing the intervention (Dempster et al., 2018; Okamoto and Hashimoto, 2022). In two studies, the non-dominant hand was used to train (Saito et al., 2021; Washio et al., 2021). One study used the domain hand to train (Mather et al., 2020). Three studies did not report relevant information (Brown and Bray, 2015; Guzmán-González et al., 2020; Yamada et al., 2021).

#### 3.4.2 Determine handgrip strength

One study determined each hand’s MVC before each training session (Dempster et al., 2018). Two studies determined MVC using the non-dominant hand (Saito et al., 2021; Washio et al., 2021). One study determined MVC using the dominant hand (Yamada et al., 2021). The remaining four studies did not report this information (Brown and Bray, 2015; Guzmán-González et al., 2020; Mather et al., 2020; Okamoto and Hashimoto, 2022).

Two studies reported the holding time used to determine the MVC. In these two studies, 5-s with a rest interval of two mins (Guzmán-González et al., 2020) and 4-s with a rest interval of one min (Brown and Bray, 2015) were used, respectively. The remaining six studies did not report this information (Dempster et al., 2018; Mather et al., 2020; Okamoto and Hashimoto, 2022; Saito et al., 2021; Washio et al., 2021; Yamada et al., 2021).

Regarding the two studies using an IHE protocol until exhaustion, one study reported the specific threshold to determine the participants’ exhaustion as a force drop greater than 10% MVC for more than 5-s (Guzmán-González et al., 2020), whereas the remaining study did not report this information (Brown and Bray, 2015).

#### 3.4.3 Assessments of cognitive function

The Memory recognition task and Go/No-Go task were adopted in two studies (Saito et al., 2021; Washio et al., 2021) and Stroop task was adopted in other two studies (Brown and Bray, 2015; Yamada et al., 2021). Trial making test (part A and part B) was adopted in two studies (Dempster et al., 2018; Okamoto and Hashimoto, 2022). Auditory oddball task was adopted in one study (Mather et al., 2020). Controlled oral word association task was adopted in one study (Dempster et al., 2018). One study adopted a proposed cognitive performance by mathematical operations in which seven have to be continuously subtracted from a random number between 300 and 700 (Guzmán-González et al., 2020).

#### 3.5 Blood pressure responses

Two studies on the chronic effects of IHE reported a significant decrease in SBP compared with baseline (Dempster et al., 2018; Okamoto and Hashimoto, 2022) and compared with the control group after the intervention (Okamoto and Hashimoto, 2022). In terms of the acute effects, one study reported a significant increase in SBP and DBP immediately following the IHE protocol (Saito et al., 2021). Another study reported that SBP and DBP were significantly higher in the exercise condition than in the rest condition and that there was a significant decrease in SBP and DBP from the exercise condition to the rest condition, but the comparison between two groups (i.e., IHE and control) was not conducted (Washio et al., 2021). The remaining four studies did not measure this variable (Brown and Bray, 2015; Guzmán-González et al., 2020; Mather et al., 2020; Yamada et al., 2021).

### 3.6 Effect of isometric handgrip exercises on cognitive function

Concerning chronic effects of IHE, two studies reported a positive effect on cognitive function, as the reaction time improved after the eight-week intervention (Dempster et al., 2018; Okamoto and Hashimoto, 2022). In two studies that adopted the exhaustion protocol for acute effects of IHE, one study reported a negative effect after the IHE (Brown and Bray, 2015). Another study reported an increased number of mathematic errors on a regulated dual-task (i.e., imposed pace) but not in the self-regulated dual-task (i.e., own pace) (Guzmán-González et al., 2020). In the four remaining studies that examined the acute effects of IHE, two studies reported positive effects on cognitive performance (Mather et al., 2020; Washio et al., 2021), and the other two studies reported no improvement in cognitive performance after the IHE intervention (Saito et al., 2021; Yamada et al., 2021).

## 4 Discussion

The current review systematically summarized the evidence concerning the acute and chronic effects of IHE on measures of cognitive performance in healthy adults. Overall, the results of this review suggest that the acute effects of IHE on cognitive function are currently inconclusive. However, this review points toward a positive chronic effect of IHE on cognitive function although this observation should be treated cautiously as only a relatively small number of studies is available. A critical methodological discussion and safety considerations are discussed below.

In studies that investigated the acute effect of IHE with different intensity levels of the intervention protocol, two studies reported a positive effect (Mather et al., 2020; Washio et al., 2021) and two other studies reported a nonsignificant effect of the intervention on cognitive performance (Saito et al., 2021; Yamada et al., 2021). Although Washio et al. (2021) and Saito et al. (2021) utilized the same task to probe cognitive performance (i.e., memory recognition task and Go/No-Go task) and the samples had comparable demographic characteristics (age = 21.6 vs. 22; male = 82% for both), the results for IHE on cognitive performance were rather inconsistent. Specifically, Saito et al. (2021) used multiple sets, short holding time, and a short recovery time protocol (i.e., 16 sets, 30-s 30% MVC with 45-s recovery) and reported a nonsignificant effect of IHE on cognitive function, whereas Washio et al. (2021) used fewer sets, longer holding time, and a longer recovery time protocol (i.e., 4 sets; 2-min 25% MVC with 3-min recovery) and observed improved processing speed in Go/No-Go task. Compared with increasing the sets for an IHE protocol, the current finding suggests that a longer holding time may induce more pronounced effects on cognitive performance. However, given the low number of available studies, further research is necessary to prove this assumption empirically.

Additionally, increasing the holding intensity (i.e., MVC) may also elicit a greater effect on measures of cognitive performance, even if the holding time is relatively short. For example, in a large sample study, Mather et al. (2020) adopted five sets, 18-s 100% MVC with 60-s recovery protocol and found that the performance of the auditory oddball task was improved immediately after IHE. Again, given the small number of available studies, no solid conclusions regarding potential dose-response relationships are possible at the moment. However, according to current evidence, the holding time of the IHE should be longer than 18 s, as the 18 s paradigm are observed to induce positive effects on cognition performance regardless of the experimental parameters (Nielsen and Mather, 2015; Mather et al., 2020).

Studies that investigated acute effects with a single bout ([one set] pre-post exhaustion protocol) reported relatively inconsistent results (Brown and Bray, 2015; Guzmán-González et al., 2020). Brown and Bray. (2015) observed that performing IHE to exhaustion was associated with impaired cognitive performance (i.e., modified Stroop task). The findings of Guzmán-González et al. (2020) suggest that such an effect is influenced by the cognitive loading during the contraction, as they observed that in response to a single bout of IHE (50% MVC to exhaustion), the cognitive performance decreased in a regulated dual-task, but was not altered in a self-regulated dual-task or no cognitive load protocol. Notably, these two studies did not include a follow-up test (e.g., 10-min after the intervention) which is a clear limitation because the relationship between exercise and cognitive function is hypothesized to be an inverted U-shaped dose-response curve (Diederich et al., 2017; Loprinzi et al., 2018). For example, a previous study found that cognitive performance was more likely to improve after a 10-min recovery following physical exercise than immediately after the cessation of the exercise program (Zhu et al., 2021). Therefore, future studies should consider implementing an appropriate follow-up test to determine the influence of exercise intensity (e.g., operationalized by % of MVC) on measures of cognitive performance.

With respect to study investigating the chronic effect of IHE, two studies performing an 8-weeks IHE (i.e., 4 sets; 2-min 30% MVC with 60-s recovery, 3–5 days/week) noticed that the older adults in the experimental group decreased their time spent in Trail Making Test, indicating an improvement of cognition processing speed (Dempster et al., 2018; Okamoto and Hashimoto, 2022). The IHE protocol employed in these two studies is comparable to the protocol used by Yamada et al. (2021), who reported only a nonsignificant acute effect of IHE on cognitive performance. Thus, even though the acute effects of IHE on specific measures of cognitive performance are not statistically significant, the findings of these two studies suggest that chronic IHE can improve cognitive performance. The latter observation is in line with the literature that blood pressure and handgrip strength are associated with cognitive function, especially in older adults (Novak and Hajjar, 2010; Fritz et al., 2017; Shaughnessy et al., 2020; Herold et al., 2021). Although the available evidence needs to be treated cautiously, the promising findings with respect to chronic effects of IHE call for future large-scale clinical trials with a rigorous study design to elucidate whether chronic IHE can be an effective intervention strategy to improve measures of cognitive performance.

### 4.1 Methodology consideration

The dose of IHE is a critical factor influencing the effectiveness of the intervention. To set an appropriate dose, a proper exercise prescription is needed (Herold et al., 2019a, 2020). With respect to IHE, our review revealed that IHE using four sets of two minutes of 30% MVC with 60-s of recovery is the most frequently applied protocol. Of note, the same protocol is also commonly used in IHE interventions that have successfully lowered blood pressure (Bakris et al., 2019; Loaiza-Betancur and Chulvi-Medrano, 2020). Given that high blood pressure is negatively correlated to brain function (Novak and Hajjar, 2010; Beauchet et al., 2013; Gąsecki et al., 2013; Ungvari et al., 2021) and cognitive performance (Novak and Hajjar, 2010; Gąsecki et al., 2013; Forte et al., 2020), a protocol that can effectively lower blood pressure might serve as a good starting point for further research aiming to elucidate the effects of IHE on the brain and cognitive performance. This is consistent with the hypothesis and evidence that isometric exercises training (e.g., IHE) plays a crucial role in the preservation of cognitive performance and prevention of Alzheimer’s disease due to their positive effects on the cardiovascular system (Hess and Smart, 2017; Okamoto and Hashimoto, 2022).

#### 4.1.1 Sets and contraction time

The high-intensity IHE (operationalized *via* MVC) has an acute and beneficial effect on cognitive performance. For example, using five sets of 18-s 100% MVC with a 60-s recovery protocol was found to improve attentional performance (Mather et al., 2020). However, there is evidence that such an IHE protocol can lead to a substantial increase in blood pressure which, in turn, may elevate the risks of adverse events. From a theoretical perspective, a safer option is to maintain the total exercise volume by decreasing the exercise intensity (operationalized by MVC) and contraction time while increasing the number of sets (Millar et al., 2011). Notably, using such a protocol with lower exercise intensity and contraction time could reduce the blood pressure response induced by IHE, but whether this has a positive effect on cognitive performance is debatable. For example, Saito et al. (2021) reported in their pilot trial that participants’ blood pressure decreased by 26% when performing IHE for 30-s (30% MVC, 16 sets, 45-s recovery, SBP = 127 mmHg, DBP = 76 mmHg) compared with the conditions in which traditional IHE was performed for 90–120 s. It should be noted, however, that cognitive performance did not change significantly when the number of sets was increased from 4 to 16 compared with a protocol with a longer contraction time (2 min, 25% MVC with 3 min of recovery) corresponding to the same cognitive tasks and population, but showed a significant improvement in cognitive performance (Saito et al., 2021; Washio et al., 2021). Based on the above-presented results, it seems feasible to improve cognitive performance by increasing the MVC and the contraction time, but it seems impossible to modulate the effect by increasing the number of sets.

#### 4.1.2 Bilateral or unilateral

Some of the reviewed studies failed to provide information on whether one (unilateral) or both (bilateral) hands were used during IHE (i.e., bilateral forms simultaneously, unilateral forms for one hand, or unilateral forms in which one hand switches to the other) (Brown and Bray, 2015; Guzmán-González et al., 2020; Yamada et al., 2021). Although no study has directly compared the difference between these two modes in terms of their effect on measures of cognitive performance, there is evidence that the physiological responses to unilateral and bilateral resistance exercises are not necessarily equal, especially during the isometric contraction (Škarabot et al., 2016). For example, McGowan et al. (2007) showed that after IHE three times over eight weeks, blood pressure reduction was significantly higher in the bilateral group than in the unilateral group (i.e., 4 sets of 2 min of 30% MVC with 1-min recovery). Additionally, the form of IHE is associated with increased experience of specific hemispheric emotional processing and cognitive processing. Specifically, following left-hand clenching, individuals became more affectively negative, and after right-hand clenching individuals experienced positive affect (Propper et al., 2017). For episodic memories, left-hand clenching (right prefrontal regions) was associated with retrieval, and right-hand clenching (left prefrontal regions) was associated with encoding (Habib et al., 2003). Whether such effects transfer or yield meaningful differences with respect to cognitive performance is currently unclear. Given that a transparent reporting of all exercise variables is mandatory to allow for a comparison across different studies and for a reproduction of the effects in research and practical settings (Gronwald et al., 2019), we strongly encourage further studies to report this information.

#### 4.1.3 Sex differences

There is some evidence that sex differences can influence the effectiveness of physical interventions (Barha et al., 2017; Barha et al., 2019; Barha and Liu-Ambrose, 2018). However, there is no report or comparison of relevant information in the reviewed studies. Among previous studies that included both males and females, inconsistent findings are reported. For example, Keller et al. (2022) used fNIRS to assess participants’ cerebral oxygenation during IHE and observed that at a 25% MVC protocol, there was a decrease in oxyhemoglobin in males but an increase in females, which directly affects cognitive performance (Herold et al., 2019b; Zhu et al., 2022). Additionally, sex differences in matched contraction load contribute to differences in BP responses and cardiac baroreflex sensitivity (Teixeira et al., 2018; Lee et al., 2021), which potentially indirectly affects cognitive performance (Ogoh and Tarumi, 2019). Lee et al. (2021) reported males having larger acute BP responses during and immediately after the 2-min with 30% MVC exercise than females. Teixeira et al. (2018) observed that for males, systolic BP increased 10 min after 2-min 30% MVC IHE and remained elevated during 20-min and 30-min, whereas in female it increased 10-min after exercise and returned to baseline during the 20- and 30-min recovery periods. Therefore, it is recommended that future studies should consider possible sex-related difference influencing the effectiveness of IHE on cognitive performance.

### 4.2 Safety consideration

Isometric resistance exercises are known to induce systemic changes in the cardiovascular system as they transiently increase systolic and diastolic blood pressure (Huggett et al., 2004). Especially the severe cardiac responses observed during high-intensity isometric contractions to exhaustion raised concerns that isometric exercise should be provided with caution in clinical patient populations, such as patients with hypertension or stroke (Saunders et al., 2014; Hanssen et al., 2022). However, several studies have reported that a single bout of isometric exercises produces equivalent, or lower, systolic BP and heart rate responses than dynamic aerobic exercise (Stebbins et al., 2002; Millar et al., 2014), and the specific effect of isometric exercise on blood pressure depends on the exercise protocol (Carlson et al., 2014; Farah et al., 2017). In general, it is recommended that IHE be performed at low-to-moderate intensity because of the lack of evidence concerning the blood pressure responses to acute isometric exercise in a variety of patient cohorts.

Moreover, the following safety recommendations should be considered (Millar et al., 2014; Allen et al., 2018) when performing isometric exercises in general and IHE in particular: 1) ensuring adequate BP responses during IHE protocols, i.e., maintaining spontaneous breathing without a Valsalva maneuver (forced expiration with the glottis closed) (Millar et al., 2014), 2) standard absolute and relative contraindications to exercise training should be taken into account (e.g., uncontrolled blood pressure >180/110 mmHg) (Millar et al., 2014), 3) while there is no golden cut-off threshold for discontinuing an exercise protocol if BP is raised too high, it is advisable to stop when the systolic BP reaches about 240 mm Hg (Fagard, 2011), 4) isometric contractions may accompany secondary symptoms such as local paresthesias and mild discomfort. This is most commonly observed towards the end of each set, especially in protocols with contractions lasting more than 2 min (Millar et al., 2014), 5) to minimize muscle damage, it is recommended to perform the hold time and MVC at the half-maximal effort or less (Allen et al., 2018), 6) adding warm-up exercises prepare the bodily systems and, in turn, can reduce the risks and adverse effects of physical exercise (McGowan et al., 2015), and 7) it is recommended to gradually increase the contraction time or MVC to achieve an appropriate load (Sun et al., 2014). Taken together, the current evidence suggests that it is possible to safely perform isometric training if the protocol is tailored to the particular clinical condition of the participant/patient (Millar et al., 2014).

### 4.3 Practical implications, limitations and further direction

Isometric handgrip exercise is a low-cost and easy-to-adopt resistance exercise that can be used in populations worldwide, especially in individuals with specific motor disabilities. In addition to the positive effect of IHE on blood pressure (Whelton et al., 2018) and pain relief (Lim and Wong, 2018) being documented in previous reviews, the results of the current systematic review provide preliminary evidence that IHE has the potential to improve cognitive function. However, the neurobiological mechanisms underlying the positive effects are still unclear and require further investigation (Stillman et al., 2016; Herold et al., 2019b). While lowering blood pressure is an important factor at least partly responsible for the benefits of IHE on cognitive function (Whelton et al., 2018), the effect on other health-related factors is still relatively unclear. In addition, exercise protocol needs to be tailored to the clinical characteristics of each population. For example, compared with aerobic exercise, IHE was ineffective on high-density lipoprotein cholesterol antioxidant function in patients with hypertension after 12 weeks of exercise (Pagonas et al., 2019). Aerobic exercise may be more suitable for populations with normal motor skills, while IHE is recommended for individuals for whom exercise with greater muscle mass is more challenging, such as patients exercising in certain locations (e.g., hospitals) or under certain circumstances (e.g., bedridden patients).

The current review has several limitations. First, due to the small number of eligible studies, the result was based on limited evidence with the narrative synthesis. A meta-analysis should be considered when more intervention studies have been conducted in this field. Second, only English-language journal articles were considered, and some potentially relevant studies published in other languages were excluded. Nevertheless, the critical methodological and safety considerations discussed in this study should serve as an important reference for future studies in research design. Some important research gaps discussed in this review also shed light on the further development of this field.

With respect to the current state of the literature, several research questions remain unanswered. Future studies should address, but are not limited to, the following research questions: 1) Are the current IHE protocols recommended to lower blood pressure also a cost-effective intervention to promote cognitive health? 2) Do IHE and dynamic handgrip exercise induce comparable effects on measures of cognitive performance? 3) Which neurobiological processes drive the positive effects of IHE on cognitive performance? Namely, 1) molecular and cellular changes, 2) functional and structural brain changes, and 3) socioemotional changes should be considered (Stillman et al., 2016; Herold et al., 2019b).

## 5 Conclusion

In conclusion, the evidence on whether IHE has acute positive effects on cognitive performance is currently rather inconclusive. However, there was a trend that implementation of IHE has a beneficial chronic effect on cognitive performance, although the results should be interpreted with caution as the findings were based on a small number of available studies and the current state of knowledge in this research area is relatively scant. Thus, further investigation with a rigorous methodological approach is needed to evaluate the feasibility and effectiveness of IHE, especially in needy cohorts such as older adults being at higher risk of cognitive decline (e.g., suffering from hypertension).

## Data Availability

The original contributions presented in the study are included in the article/Supplementary Material, further inquiries can be directed to the corresponding author.
