# Supplementary material for: Effect of isometric handgrip exercise on cognitive function: Current evidence, methodology, and safety considerations
Source: Front Physiol. 2022 Oct 4;13:1012836. doi: 10.3389/fphys.2022.1012836 (PMC9576950; doi:10.3389/fphys.2022.1012836)
Supplement: Supplementary file 1 [file Table1.DOCX]

Table S1: Risk of bias of included studies (“0” = No, “1” = Yes)

| First author | 1 | 2 | 3 | 4 | 5 | 6 | 7 | 8 | 9 | 10 | 11 | Total^*^ |
| --- | --- | --- | --- | --- | --- | --- | --- | --- | --- | --- | --- | --- |
| Washio et al., 2021 | 1 | 1 | 1 | 1 | 0 | 0 | 0 | 1 | 1 | 1 | 1 | 7 |
| Yamada et al., 2021 | 1 | 1 | 1 | 1 | 0 | 0 | 0 | 1 | 1 | 1 | 1 | 7 |
| Saito et al., 2021 | 1 | 1 | 1 | 1 | 0 | 0 | 0 | 1 | 1 | 1 | 1 | 7 |
| Mather et al., 2021 | 1 | 1 | 1 | 1 | 0 | 0 | 0 | 1 | 1 | 1 | 1 | 7 |
| Brown et al., 2015 | 1 | 1 | 1 | 1 | 0 | 0 | 0 | 1 | 1 | 1 | 1 | 7 |
| Guzmán-González et al., 2020 | 1 | 1 | 1 | 1 | 0 | 0 | 0 | 1 | 1 | 1 | 1 | 7 |
| Dempster et al., 2018 | 1 | 0 | 1 | 0 | 0 | 0 | 0 | 1 | 1 | 0 | 1 | 4 |
| Okamoto et al.,  2022 | 1 | 1 | 1 | 1 | 0 | 0 | 1 | 1 | 1 | 1 | 1 | 8 |

*Note*: * Each satisfied item (except item one) contributes one point to the total score
